# Application of engineered extracellular vesicles for targeted tumor therapy

**DOI:** 10.1186/s12929-022-00798-y

**Published:** 2022-02-21

**Authors:** Fusheng Zhang, Jinshuai Guo, Zhenghou Zhang, Meiqi Duan, Guang Wang, Yiping Qian, Haiying Zhao, Zhi Yang, Xiaofeng Jiang

**Affiliations:** Department of General Surgery, Fourth Affiliated Hospital of China Medical University, Shenyang, China

**Keywords:** Extracellular vesicles, Drug delivery system, Targeted tumor therapy, Mesenchymal stem cells

## Abstract

All cells, including prokaryotes and eukaryotes, could release extracellular vesicles (EVs). EVs contain many cellular components, including RNA, and surface proteins, and are essential for maintaining normal intercellular communication and homeostasis of the internal environment. EVs released from different tissues and cells exhibit excellent properties and functions (e.g., targeting specificity, regulatory ability, physical durability, and immunogenicity), rendering them a potential new option for drug delivery and precision therapy. EVs have been demonstrated to transport antitumor drugs for tumor therapy; additionally, EVs' contents and surface substance can be altered to improve their therapeutic efficacy in the clinic by boosting targeting potential and drug delivery effectiveness. EVs can regulate immune system function by affecting the tumor microenvironment, thereby inhibiting tumor progression. Co-delivery systems for EVs can be utilized to further improve the drug delivery efficiency of EVs, including hydrogels and liposomes. In this review, we discuss the isolation technologies of EVs, as well as engineering approaches to their modification. Moreover, we evaluate the therapeutic potential of EVs in tumors, including engineered extracellular vesicles and EVs' co-delivery systems.

## Highlights

1. Technologies such as microfluidics can improve EVs isolation efficiency.
2. Engineering technologies can improve EVs drug loading efficiency and tumor targeting.
3. EVs-based drug co-delivery systems are being developed, such as those with liposomes and hydrogels.

## Introduction

Tumors have been responsible for numerous deaths in the last decades. Owing to high recurrence and mortality rates, drug resistance, metastatic ability, and poor prognosis, they continue to pose a serious threat to human health [1]. Despite the immense advances in medical technology over the years, the clinical outcomes of tumor treatment remain unsatisfactory, owing mainly to the lack of site-specific drug targeting capacity, which results in suboptimal chemotherapeutic outcomes [2]. Although chemotherapy is one of the main treatments for tumors, it also has significant limitations, including low delivery efficiency, tissue resistance, and insufficient drug targeting ability [3]. Additionally, low permeability and poor bioavailability in body fluids also limit the efficacy of chemotherapeutic drugs. These observations highlight the urgent need for the development of new drugs and drug delivery systems that are efficient, safe, and can be targeted to tumors.

Over recent years, numerous clinical trials and studies have demonstrated that extracellular vesicles (EVs) can serve as excellent drug carriers and have the potential to improve therapeutic effects against tumors [4]. They contain a large variety of genetic information and are widely present in blood, saliva, urine, tears, and cerebrospinal fluid, among other body fluids [5]. Additionally, because they are secreted by cells, EVs are biosafe, stable, and have good target specificity [6, 7]. They also have the advantages of deep tissue penetration and a surface structure that is similar to that of the cell membrane [6, 8], which allow them to act as carriers for the delivery of drugs to sites of disease [7]. Given their unique biological behavior, EVs have enormous potential for using in immunotherapy and precision treatment. With the advent of technologies that allow their modification, such as loading contents, EVs can now be modified to further improve their efficiency of drug delivery and target specificity, thus also enhancing therapeutic outcomes for tumor patients [9, 10]. However, the diversity of cell differentiation statuses and EVs composition has resulted in a lack of standardized isolation techniques and modification methods for EVs [11]. “Exosomes” are small membrane vesicles that contain a variety of substances and are a type of EVs [12]. However, only few studies have proven that the therapeutic EVs are exosomes, we refer to exosomes as “extracellular vesicles” throughout the review. Here, we focus on the isolation technologies of EVs, methods for their modification, and the therapeutic potential of EVs-based drug delivery systems.

## Biological properties

EVs are rich in cholesterol, sphingomyelin, microRNAs (miRNAs), long non-coding RNAs (lncRNAs), a multitude of proteins that participate in intercellular communication [13, 14]. Importantly, cargo carried by EVs can play key pathophysiological roles, including via intercellular communication and immunological responses [13, 15]. EVs are nanoscale vesicles enveloped by cyto-membranes and can be broadly categorized into three types based on size: apoptotic vesicles, which are > 1000 nm in diameter [16]; microvesicles, which range from 100 to 1000 nm in diameter [17]; and exosomes, which have diameters that vary between 30 and 100 nm [18]. Moreover, EVs can be isolated and purified by using a variety of methods, including ultracentrifugation and chromatographic columns [19].

## EVs acquisition

EVs can be broadly classified into three subtypes based on their origins: cell culture-derived EVs, bodily fluid-derived EVs, and tissue-derived EVs [20]. Nevertheless, because EVs are secreted by cells into body fluids and tissues, cellular secretion represents the primary source of these vesicles.

For cell culture-derived EVs, the cell culture medium is a critical determinant of EVs yield as it supports cell proliferation and expansion. Three distinct types of medium are currently used to obtain cell culture-derived EVs: serum-containing medium, serum-free medium, and chemical composition substitution [21]. Notably, serum-free medium is frequently supplemented with components such as human platelet lysate (HPL) [22]. In general, serum-containing media are more conducive for cell growth and enhanced EVs production compared with serum-free media. HPL, fetal bovine serum (FBS), and human serum are frequently included in serum-containing media, and these media promote considerable cell proliferation [23, 24].

Importantly, when cells or products are employed for medicinal purposes, media containing animal components, such as FBS, should be avoided. Although information regarding serum concentrations in cell culture media is limited, higher serum concentrations are not always better, and should thus be kept within a tolerable range; however, this can vary according to the cell line used for EVs production [25]. Generally, serum-free media have less protein, which is detrimental to cell proliferation. Scaling up cell culture in such media typically requires supplementation with additives, cell growth components, and proper media adjustment procedures, known as chemical composition substitution [26]. Therefore, either medium with a complex chemical composition must be produced to allow for scale expansion, or the initial number of cells must be increased to critical levels in another medium before the cells are transferred to this serum-free medium for EVs production [21]. Additionally, studies have been reported on the effect of environmental conditions on the media, with oxygen content (reduced from the normal 21 to 2–7%), shear stress, and stimulation with proinflammatory cytokines (IFN-γ and TNF-α) all reported to enhance the quality or quantity of secreted EVs [27, 28]. Environmental conditions are difficult to standardize for the industrial-scale production of EVs because EVs production varies by cell.

Compared with cell culture, body fluids contain mixtures of broader origin, such as serum proteins or mixtures of systemic EVs, which complicates their isolation [29]. Furthermore, the proportions of EVs in the circulatory system that are released from specific tissues are unknown [30]. Accordingly, cell culture is more frequently used to obtain EVs than bodily fluids. EVs in body fluids are increasingly being utilized for disease diagnosis and prognosis based on the surface protein profile of the EVs in each sample [31]. Research interest in the use of tissue-derived EVs (Ti-EVs) has markedly increased owing to several advantageous characteristics. Importantly, tissues contain vesicles released by most cell types in the tissue microenvironment and, thus, more precisely reflect pathological features [29]. Moreover, because Ti-EVs are produced from a single tissue, they have fewer impurities compared with bodily fluid-derived EVs [32]. However, other challenges, such as obtaining a therapeutic-scale yield, currently limit the use of Ti-EVs. Feasible solutions for this limitation include the reconstitution of 3D tissue-like structures (organoids), bioreactor-based culture methods, and treatment efficiency enhancement [20]. However, these technologies are still in their infancy, and require substantial research attention. Cell incubation remains the most frequently used approach for producing EVs on a large scale. However, there seems to be few devices have been developed for obtaining EVs through large-scale cell culture, a drawback that requires research attention.

## Techniques for EVs isolation

Further assessment of EVs properties and uses requires the isolation of EVs of greater purity, i.e., cell debris and other interfering components must be removed. EVs secreted by different cells vary in size, function, and content, posing challenges to their extraction and isolation [33]. However, different EVs isolation and purification methods can be employed according to their characteristics [34, 35], including size, shape, density, and surface receptors (Table 1). The most common of these methods are ultracentrifugation, size-based isolation techniques, polymer precipitation techniques, immunoaffinity chromatography, and microfluidic-based isolation techniques.

**Table 1:** Summary of extracellular vesicles isolation techniques

Technique	Principle	Advantages	Disadvantages	Isolation capacity	Purity	Time required	References
Ultracentrifugation (UC)	Particles of different sizes and densities are separated using different speeds	•Gold standard for isolation •Suitable for large-volume samples	• Dependence on equipment • Low purity • Protein aggregation • EVs can be destroyed by high-speed centrifugation	Low	Low	Medium	[37, 38, 196]
Density gradient centrifugation	Using differences in particle density to maintain different particles in the proper density medium and perform centrifugation	• The purity of EVs is higher than that obtained through UC • Applicable to the isolation of EV subpopulations	• Dependence on equipment • Time-consuming • Risk of EV destruction	Medium	Medium	High	[37, 38, 44]
Ultrafiltration	Utilizes differences in EV particle size and molecular weight	• Easy to operate • Direct extraction of RNA • Low equipment cost • Good portability	• Insufficient specificity • May contain impurities • Moderate purity	Medium	Medium	High	[57, 197]
Size exclusion chromatography (SEC)	Utilizes differences in EV particle size and molecular weight	• Preservation of EV structural integrity • Easy to operate • Higher purity than that obtained through UC	• Time-consuming • Possibility of pore blockage • May contain impurities • High device costs	Low	Medium	High	[40, 52, 198, 199]
Polymer precipitation	Precipitation of EVs through reduced polymer solubility	• Equipment-independent method • High efficiency •Can be used for large samples • Easy to operate	• Easy of contamination • Insufficient specificity • Protein aggregation	Low	Medium	High	[50, 54, 200]
Immunoaffinity chromatography	Specific antibodies bind to proteins on the surface membrane of EVs	• Higher purity than that obtained through UC • No chemical contamination • Suitable for isolating EVs with identical membrane proteins	• High cost • Can influence EV activity • EV markers must be optimized • Unsuitable for isolation from large samples	Medium	Medium	High	[61, 196, 201]
Microfluidic technology	Microscale technique using equipment based on physicochemical differences in EVs	• Simplicity and efficiency • Ease of automation and integration • High sensitivity and higher purity compared with that obtained through UC	• Requirement for complicated equipment • Lack of uniform standards	High	Medium	High	[66, 201, 202]

### Centrifugation-based techniques

Ultracentrifugation is considered the “gold standard” and is the most widely used technique for EVs isolation. The principle of ultracentrifugation is simple and mainly relies on differences in size, shape, and density of the components in the sample to obtain EVs under the appropriate centrifugal force [36]. The samples are subjected to multiple low-speed centrifugations to remove large biological particles, such as cell debris and apoptotic vesicles, and then centrifuged at high speed, following which the EVs contained in the supernatant are collected [36, 37]. This method is suitable for the isolation of samples with large sedimentation coefficients. Although this technique is widely used owing to its simplicity, it is time-consuming. Moreover, the centrifugal force and rotor type can affect EVs purity, and the acquisition efficiency may not be sufficiently stable. Also, repeated centrifugation may result in EVs structural damage, thereby affecting their quality, which is not conducive to their subsequent analysis [37, 38]. This was shown in a study of supernatants obtained from non-small cell lung cancer cells (SK-MES-1), where EVs quality and recovery rate were reported to be reduced after repeating ultracentrifugation [39].

Density gradient centrifugation can yield EVs of higher purity compared with ultracentrifugation [40]. As the name implies, density gradient centrifugation separates the sample components based on differences in density, with greater differences indicating more effective isolation [41]. First, biological media of different densities (e.g., sucrose or glycerol iodide) are placed in a test tube, with the density of the media increasing sequentially from top to bottom. Next, the target sample is added to the top of the media and centrifuged with the appropriate force for the appropriate duration. Eventually, extracellular components are obtained in a layer with a specific density [42, 43]. Despite its simplicity and high-purity yield, this is a time-consuming and instrument-dependent method [44]. Additionally, EVs quality can be affected by the duration and force of centrifugation, which limits the large-scale application of this approach [37, 38].

### Size-based isolation techniques

Size-based isolation techniques mainly refer to ultrafiltration and molecular exclusion chromatography, which are used to isolate EVs based on differences in size and molecular weight.

In the ultrafiltration technique, ultrafiltration membranes with different degrees of molecular weight retention are used to isolate EVs [45]. This method is suitable for the isolation of particles < 100 nm in diameter and can serve as an alternative to ultracentrifugation for EVs isolation owing to its higher isolation efficiency [46]. Furthermore, the employment of ultrafiltration membranes and columns with pore diameters of varying size allows for creating a population of particular dimensions, and plasma EVs of specific diameters have been obtained using such columns [47, 48]. Notably, however, ultrafiltration is a time-consuming process, the pores in the ultrafiltration membrane are easily clogged, and the pressure applied during isolation may deform or rupture the EVs [49]. Sequential centrifugation has been proposed as a means of improving the ultrafiltration method. This involves the removal of impurities (e.g., cell debris) using a slightly larger pore size, followed by the depletion of free proteins, sample concentration, and, finally, EVs isolation using an ultrafiltration membrane with a specific pore size [50, 51]. Sequential centrifugation has been used to isolate EVs of high purity from human colon cancer cell lines [51].

The chromatographic method is based on the pore size of the stationary-phase gel of the isolation column. Sample components with higher molecular weight cannot enter the gel and are eluted early, while those with lower molecular weight (e.g., EVs) enter the pores and are eluted late [52]. This method is simple and retains the biological activity and structural integrity of EVs. Nevertheless, numerous disadvantages (e.g., the requirement for an isolation column, the laboriousness of the process, the column is easily contaminated by impurities, and the high cost) limit the application of this method [40, 52].

### Polymer precipitation

Polymer precipitation is a commonly used technique for EVs isolation. Polyethylene glycol (PEG) interacts with water molecules near EVs to form a specific environment, which reduces the solubility of the EVs and causes them to precipitate [50]. The samples are pretreated to remove contaminants and subsequently co-cultured with PEG solution under suitable conditions for 24 h [53]. Finally, the precipitates are centrifuged to obtain EVs. The polymer precipitation method is simple, rapid, cost-effective, and not dependent on expensive equipment, meaning that it can be utilized for the large-scale isolation of EVs. However, polymers can also precipitate proteins, nucleic acids, and lipids and result in EVs contamination [54], thereby reducing EVs isolation efficiency and affecting their analysis. Commercial kits that employ polymer precipitation techniques for the isolation of EVs are currently available. Nonetheless, with this method, protein and lipid precipitation can contaminate EVs samples. Additionally, the high cost of polymer precipitation processes limits their applicability.

### Immunoaffinity chromatography

Numerous specific membrane proteins are present on the EVs membrane surface, including CD63, HSP20, and chaperonin containing TCP1 subunit 2 (CCT2) [55, 56]. These surface proteins can be used as specific molecular markers for EVs isolation. Immunoaffinity chromatography is used for the isolation and purification of EVs through specific antibody/ligand binding [57]. The isolation efficiency is closely related to protein affinity, matrix carriers, and elution conditions. This method can be used for the qualitative and quantitative analysis of EVs. According to the antibody substrate, this technique is mainly divided into the magnetic bead method, chromatographic fixation method, and enzyme-linked immunosorbent assay. Immunoaffinity chromatography offers the advantages of high specificity, high sensitivity, high EVs purity, and the maintenance of morphology [58]. Quantitative assays have shown that, compared with ultracentrifugation, EVs purity can be enriched using this approach through the binding of the Vn96 peptide to EVs-resident heat shock proteins [59]. Because this technique uses EVs surface proteins for isolation, it can be used to isolate subpopulations of EVs produced by specific cells, thereby allowing for in-depth studies on their biological properties and functions. To date, immunoaffinity chromatography has been used to isolate EVs derived from T cells and melanoma cells. Using CD63 aptamers, Song et al. demonstrated that this technology might increase the efficiency of EVs isolation and lead to improved cancer diagnosis [60, 61]. Additionally, the affinity of antibodies for their ligands enables them to access EVs-specific populations. Thus, specific EVs populations can be isolated using CD9-antibody-immobilized immunoaffinity and CD63 aptamer immunoaffinity [62].

This approach also has some disadvantages, including high cost, low yield, a requirement for strict storage conditions, and the susceptibility of EVs activity to changes in pH and salt concentrations [62, 63]. In addition, immunoaffinity chromatography is unsuitable for the isolation of EVs from large samples, and may also affect EVs purity through the adsorption of impure proteins, thereby limiting the applicability of this technique. Moreover, more stable and inexpensive antibody alternatives can be adopted to overcome the disadvantages of immunoaffinity chromatography, such as aptamer technology using RNA for the specific recognition of EVs [64]. The advantages of this technology are low variability, low cost, low immunogenicity, and ease of chemical modification. The stability of the technology depends on factors such as temperature, ionic strength, and buffer used [65].

### Microfluidics-based isolation techniques

Microfluidics is an emerging technology used for the isolation of EVs from nanoparticles. Microfluidic-based methods can accurately and rapidly isolate EVs and improve enrichment efficiency [66, 67]. Consequently, they show promise for large-scale EVs isolation. Currently, the most widely used microfluidic techniques are primarily based on EVs particle size and immunoaffinity chromatography. Several devices have been developed for EVs isolation (e.g., acoustic, electrophoretic, and electromagnetic manipulation) [68–70]. A sonar filter is used to apply ultrasonic radiation to the sample particles. Because of differences in size and density among the particles, larger particles are subjected to stronger ultrasonic forces and thus migrate faster to the pressure node to complete EVs isolation [70]. Biocompatible polymers can also be added to the culture medium to control the viscoelasticity, yielding EVs of higher purity in the supernatant or serum [68]. In addition, microfluidic chips with an antibody coat have been used to efficiently isolate EVs from plasma [71]. Microfluidic technology provides excellent control of sample flow and mixing rate, rendering it an ideal tool for studying nanoparticles such as EVs [72]. Microfluidics can be used to isolate various EVs populations based on differences in particle size and immunoaffinity. This technique is analogous to the size-based isolation and immunoaffinity chromatography procedures previously discussed. However, as microfluidic technology advances, more suitable procedures and devices for EVs isolation will likely be developed.

Owing to their role as transmitters of information between cells, EVs have promising potential as carriers of specific drugs and as a source of diagnostic and prognostic markers. The EVs subtypes have distinct modes of biogenesis, organelle sources, and compositional components.The comprehensive isolation and description of the spectrum of EVs subpopulations from a given source are required for a thorough understanding of their constituent molecules. Additionally, a high degree of purification of the vesicular populations is required to allow the characterization of the functional and therapeutic potential of EVs. Centrifugation is currently the most frequently used approach for EVs isolation. Nevertheless, techniques such as microfluidics and immunochromatography also show potential, but require optimization. Superior isolation procedures produce vesicles of high purity and consistent particle size and limit batch-to-batch variance among EVs subpopulations, which further facilitates EVs engineering and enhances the stability and scalability of therapeutic EVs. Additionally, the scalability of EVs production allows for increased drug transportation for targeted tumor therapy.

## Engineering modifications of EVs

**Fig. 1:**
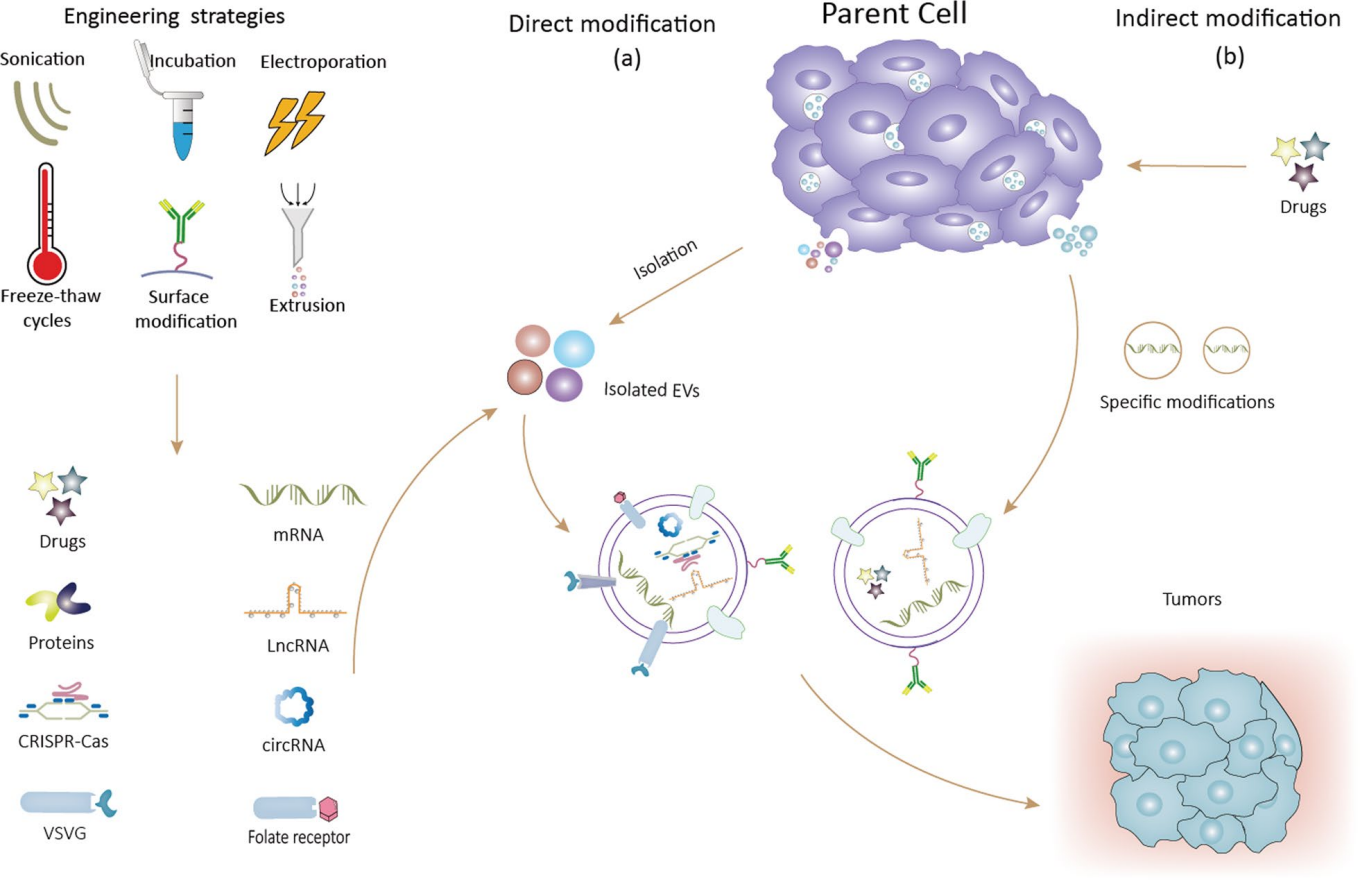
Strategies for engineering extracellular vesicles (EVs), including direct and non-direct modifications. **a** Modification performed by the loading of drugs, RNAs, etc. **b** Modification through interference with parental cells

Because of their excellent properties, including biosafety, stability, and target specificity, EVs are considered to be good drug carriers for tumor therapy and represent a new generation of nanoscale drug delivery systems [6, 17]. However, the use of EVs for tumor therapy has several limitations, such as inefficient drug delivery and inadequate targeting ability. Over recent years, EVs have been specifically engineered to improve their tumor-targeting ability and drug delivery efficiency (Fig. 1).

### Direct modifications

Direct modification, also termed “EV engineering”, the modification of surface proteins or contents of purified EVs using specific techniques under suitable conditions. This process is mainly divided into physical and chemical alterations.

#### Physical modifications

Owing to their structural characteristics, the surface and contents of EVs can be modified by physical means. The currently available methods include liposome membrane fusion and EVs content loading.

##### Surface modifications

The traditional surface modification approach involves the expression of polypeptides or proteins on the EVs membrane using specific means. The sequences of peptides and targeted proteins have been inserted into EVs-associated membrane proteins to improve tumor targeting [73]. For instance, EVs containing imatinib have been fused to IL3 receptor to target chronic granulocytic leukemia and inhibit tumor growth [74]. Because EVs are enclosed in a lipid membrane, they can be fused with liposomes to form hybrid EVs [75]. Moreover, lipids with different compositions can affect the interactions between engineered EVs and cells. To obtain EVs with functional ligands, it is first necessary to prepare liposomes with the desired ligand and to complete the engineering of EVs through cell membrane fusion [76]. Liposomes were fused with mesenchymal stem cell-derived EVs to obtain a hybrid liposome system that was then loaded miR-34a. This system was shown to significantly enhance miRNA delivery efficiency and delay the progression of breast cancer [77].

Glycosylation is a stable EVs surface peptide modification that protects EVs surface proteins from hydrolytic degradation by proteases and can act to target EVs to specific tissues. Glycosyl phosphatidylinositol (GPI) is used for EVs surface modification and can serve as an anchor for functional ligands on the EVs surface [78]. GPI is suitable for the anchoring of a wide range of functional ligands, including proteins, antibodies, and RNA. Glycosylation is thought to improve EVs-based tumor therapy by enhancing targeted polymorphic effects [79]. Hong et al. engineered enzymatic vesicles expressing the native GPI-anchored form of interleukin-2 (IL-2) to improve tumor therapeutic efficiency through the targeting of the immunosuppressive and therapy-resistant tumor microenvironment (TME) [80].

##### Modifications of EVs contents

Targeting and drug transport efficiency can also be improved by modifying EVs contents through other means (Table 2), such as electroporation. The established content modification process involves the loading of EVs with chemotherapeutic drugs, proteins, miRNAs, and lncRNAs.

**Table 2:** Direct physical modification of extracellular vesicles (EVs) (post-isolation and drug-loading modifications)

Modification method	Advantages	Disadvantages	Drug delivered	Drug delivery efficiency	Application	References
Simple incubation	• Simplest operation •Device-independent method	• Low loading efficiency • Time-consuming	PTX, DOX, porphyrins, lncRNA	Approximately 15%	Loading EVs with drugs	[83, 203]
Electroporation	• Ability to load large molecules • Higher efficiency compared with simple incubation	• Disruption of EV integrity and induction of siRNA aggregation • Device-independent method	DOX, PTX, miRNA, porphyrins	Approximately 20%	Drug loading and targeting enhancement	[204–206]
Sonication	• High efficiency • Suitable for small mRNA	• Device-dependent method • Destruction of the stability of EVs membrane	PTX, DOX, miRNA, siRNA	Approximately 25%	Improvement of drug loading efficiency	[91, 92, 207]
Extrusion	• Easy of operation • High efficiency • Short duration	• Device-dependent process • Disruption of the EV membrane	Catalase, DOX	Approximately 23%	Improvement of drug delivery efficiency and activity against tumor tissue	[83, 92]
Freeze–thaw	• No change in EV surface charge	• Low loading efficiency owing to EV aggregation	Porphyrins, PTX	High drug delivery capacity	The targeting of tumors with loaded drugs	[94–96]
Saponin	• Higher loading efficiency compared with simple incubation	• Possible membrane degeneration • In vivo toxicity	DOX, porphyrins	Approximately 15%	Loading EVs with drugs and enhancing antitumor effects	[96, 208]

*DOX* doxorubicin, *lncRNA* long non-coding RNA, *miRNA* microRNA, *PTX* paclitaxel, *siRNA* short interfering RN

*Simple incubation *The co-incubation of EVs with drugs at room temperature is a commonly used method for drug loading. Although this approach is simple, it is inefficient mainly because the EVs volume and small membrane pore size limit drug entry [81]. The clustered regularly interspaced short palindromic repeats (CRISPR)/CRISPR-associated protein (CRISPR-Cas) system can be delivered to EVs secreted by mesenchymal stem cells (MSCs) through a simple liposome/EVs incubation process [82]. However, the loading of EVs with drugs through simple incubation is inefficient when compared with electroporation, extrusion, and sonication [83].

*Electroporation* The EVs surface has numerous porous channels and charge. Hence, incubation with drugs and the application of an electric current can be used to instantly improve the permeability of the EVs membrane, allowing for rapid drug entry [84, 85]. Nonetheless, electroporation can induce vesicle or siRNA aggregation, thereby damaging the structure of engineered EVs or reducing the therapeutic effect of loaded drugs [86]. To overcome this challenge, trehalose pulse medium or chelating agents such as ethylenediaminetetraacetic acid can be added to the reaction [87]. Furthermore, one study reported that, for the loading of miRNA-155 into EVs by electroporation, the best loading efficiency was achieved at a voltage of 0.13–0.2 kV and an EVs concentration of 500–1000 mg/mL [88].

*Sonication* In sonication, an ultrasound probe with different amplitudes is used to permeabilize the EVs membrane and promote drug loading [89]. Sonication resulted in better drug loading efficiency and slower release compared with other physical methods such as simple incubation and electroporation [83]. Moreover, sonicated EVs display better biocompatibility and targeting effects. However, ultrasound radiation promotes the aggregation of EVs surface proteins and affects the physicochemical properties of the EVs membrane. Notably, sonication destabilizes the structure of EVs to a greater extent than other physical methods [90]. The loading of paclitaxel (PTX) into macrophage-derived EVs through sonication led to a > 50-fold increase in the cytotoxicity of the drug, resulting in significant inhibition of tumor tissue growth in vivo [91].

*Extrusion* In this method, an extruder is used to squeeze the cells co-cultured with the drug to complete drug loading. This approach results in uniformly sized EVs and more efficient drug loading compared with simple incubation [92]. For instance, EVs mimics were obtained by multiple sequential extrusion of MCF10A cells, and subsequently encapsulated with siRNA. The drug-loading efficiency of EVs mimics was reported to be higher than that obtained with natural EVs [93]. Importantly, mechanical extrusion can affect EVs membrane integrity, indicating that extrusion conditions and environment should be taken into consideration when this method is employed for EVs loading [89].

*Freeze–thaw cycles* Freeze–thawing involves the formation of temporary pores on the EVs membrane through multiple rapid freeze–thaw cycles to allow drug entry [94]. Freeze-thawing can be applied in the mass production of EVs; however, the associated loading efficiency is lower than that observed with ultrasonication [91]. Repeated freeze–thaw cycles can lead to an increase in EVs particle size and may improve drug loading efficiency [95].

*Saponin* Saponins are surfactant molecules that, when incubated with EVs, form pores in their membranes through interaction with cholesterol. This process increases membrane permeability, thereby improving drug loading. After drug loading, the EVs are purified by membrane dialysis and chromatography [96]. This method allows gentle drug loading into EVs without disrupting the integrity of the membrane surface, resulting in high loading efficiency and stable drug release [83]. Saponin-mediated permeabilization has been used to load hydrophilic porphyrin, leading to an 11-fold higher efficiency of drug loading into breast cancer cell-derived EVs relative to the passive loading method [96].

*Virus loading* The loading of EVs with specific drugs can be achieved using viruses. This approach can increase the efficiency of transport of certain cargos by EVs, such as RNA and proteins [97–99]. One study demonstrated that infection with Zika virus resulted in more efficient RNA and protein packaging, as well as a better transport rate, in neuronal cell-derived EVs [99]. Furthermore, “engineered EVs” formed by the fusion of the MS2 phage shell protein with EVs-associated proteins led to a six-fold increase in RNA loading efficiency [98].

#### Chemical modifications

Chemical modification mainly involves the transformation of the EVs surface and can be categorized as covalent and non-covalent (Table 3). Covalent modifications can be accomplished by chemical reactions involving EVs and specific molecules or chemical linkers. Notably, non-covalent modifications can be accomplished by electrostatic interactions and lipid fusion under suitably mild conditions.

**Table 3:** Direct chemical modification of extracellular vesicles (EVs) (post-isolation modification)

Modification method	Source of EVs	Strategy	Drug delivery	Application	References
Electrostatic interactions	HeLa cells	A complex formed by a cationic lipid and a pH-sensitive fusion peptide binds EVs through electrostatic interactions	Dextran, saponin	Targeting the cell membrane receptor to enhance cell uptake and release of EVs	[209]
Ligand–receptor interaction	Embryonic stem cells	The DSPE-PEG2000-cRGDyK targeting peptide is prepared by chemical reaction; subsequently, the ligand is inserted into the extracellular lipid bilayer through hydrophobic interaction	PTX	Penetration of the blood–brain barrier and the targeting of glioblastoma to inhibit tumor cell activity	[210]
Chemical reaction	Not mentioned	The coupling of EV azide lipids to target peptides using copper-free catalytic click chemistry	PTX, TPZ	Increasing the targeting of tumor tissues by EVs	[76]
Loading peptide for EVs	Tu-EVs	Covalently linking the functional N-terminal domain of HMGN1 (N1ND) to CP05	NA	Enhancement of the antitumor effect by increasing the ability of dendritic cells to stimulate T cells	[108]
Loading nucleotide sequences	Liver cancer cells (HepG2 cells)	Combination of molecular recognition between aptamer nucleotide sequences and their molecular targets with aptamer-chimeric trigger	NA	EV modification and functionalization, holds promise for a wide range of biomedical and bioanalytical applications	[211]

*HMGN1* high-mobility group nucleosome binding domain 1, *NA* not applicable, *TPZ* tirapazamine

##### Covalent modifications

Covalent modifications mainly include chemical conjugation, which is the direct attachment of ligands to the EVs surface through click chemistry. The numerous amino groups on the EVs surface can be used as targets for simple molecules such as fluorescent dyes and PEGs [100]. The surface modification of EVs through the azide–alkyne cycloaddition reaction has been achieved, resulting in the conjugation of azide-fluor 545 to EVs chemically modified with alkyne groups [101]. In addition, strain-promoted alkyne–azide cycloaddition between dibenzocyclooctyne and azide-labeled cRGD peptide on the surface of EVs can be targeted to treat ischemic injury in the mouse brain [102]. Chemical conjugation is characterized by speed of reaction, high selectivity, and compatibility. Furthermore, this method does not affect the structural integrity of EVs. However, conditions (e.g., temperature, pressure, and osmotic pressure) must be carefully controlled during the modification process to avoid EVs rupture and denaturation [103].

##### Non-covalent modifications

The genetic engineering of EVs-producing cells and click chemistry are both widely used for EVs surface modification. Certain non-covalent modification techniques are also under investigation for the production of targeted EVs, such as electrostatic and ligand–receptor interactions.

*Electrostatic interactions* The EVs surface is negatively charged with a zeta potential of approximately − 8.82 mV. This can be used to equip the EVs with cations, which is achieved by the binding of high-valent cations to the negatively charged EVs surface [104]. Accordingly, an EVs-based immunoblocker was designed to enhance the phagocytosis of tumor cells by macrophages through antagonism of the interaction between CD47 and SIRPα [105, 106]. However, some cationic materials may be cytotoxic and can cause lysosome degradation and reduce EVs purity when they enter the cell [107].

*Ligand–receptor interactions *This method uses hydrophobic ligands or lipid ligands for automatic insertion onto the EVs surface through hydrophobic interaction. PEG-modified liposome derivatives are commonly mixed with EVs and incubated at specific temperatures to automatically insert the liposomes onto the EVs surface. The modification of EVs derived from mouse neuroblastoma cells with PEG significantly prolonged the EVs circulation time in blood and improved the targeting of EVs.

*Others* Certain peptides and nucleotide sequences can be loaded onto the EVs surface through specific approaches. For example, the CP05 peptide was loaded into EVs after binding to N1ND. This process enhanced the ability of dendritic cells (DC) to stimulate T cells for tumor immunotherapy [108].

### Indirect modification

Although most cells can produce EVs, those released by parental cells can differ depending on the culture conditions [109]. Therefore, incubating parental cells under the appropriate conditions allows for the generation of specific EVs types. The parental cells of EVs can be genetically and metabolically engineered to enhance the tumor-targeting capabilities and drug delivery efficiency of the derived EVs (Table 4).

**Table 4:** Indirect modification of extracellular vesicles (EVs) (pre-isolation modification)

Modification method	Parent cells	Strategy	Drug loaded	Application	References
Genetic engineering	Dendritic cells	Fusion of Lamp2b-expressing engineered mouse immature dendritic cells with the IRGD peptide to produce tumor-targeting EVs	DOX	Targeting of tumor tissue and inhibition of tumor growth	[203]
	HEK293 cells	Donor cells were designed to express the transmembrane region of the platelet-derived growth factor receptor fused to Ge11 peptides to achieve tumor targeting treatment	Let-7a miRNA	Enhancement of tumor targeting and the antitumor effect of EVs	[212]
Metabolic engineering	B16F10 cells	Combining metabolic markers of newly synthesized proteins or glycoproteins from EV-secreting cells with reactive azide and bio-orthogonal click splicing	Streptavidin–HRP	Delivery of various anti-biotin protein fusions or biotin-coupled drugs	[113]
Membrane engineering	Not mentioned	Coupling of EVs containing azide lipids to targeted peptides by copper-free click chemistry	PTX	Enhancing the targeting effect of EVs against cancer cells	[76]
Loading contents in parent cells	HEK293T cells	The gene encoding pre-miR-199a was inserted into an artificial intron of the Lamp2a fusion protein. Enhanced the EV load of pre-miR-199a containing a modified TAR RNA loop using TAT peptide/HIV-1 TAR RNA-interacting peptide	miRNA	Improvement of the drug delivery efficiency of EVs	[114]

*DOX* doxorubicin, *EV* extracellular vesicle, *HIV* human immunodeficiency virus, *HRP* horseradish peroxidase, *IRGD* internalizing RGD, *Lamp2b* lysosome-associated membrane glycoprotein 2b, *miRNA* microRNA, *PTX* paclitaxel, *TAR* trans-activation response, *TAT* trans-transcriptional activator

#### Genetic engineering

Genetic engineering for the modification of parental cells to achieve improved targeting and a greater number of drug-loaded EVs is becoming increasingly sophisticated. Membrane proteins on the surface of EVs can bind to target ligands. Accordingly, a plasmid vector encoding the membrane protein Lamp2b fused to the rabies viral glycoprotein (RVG) was transfected into parental cells. Subsequently, parental cells produced EVs expressing the Lamp2b/RVG fusion as a targeting peptide on the EVs surface [110]. Lamp2 is the commonly used targeting membrane protein. For example, the cardiac-targeting peptide/Lamp2b fusion protein was transfected into HEK293 cells to obtain EVs that could target the heart [111]. Alternatively, DCs were transfected with a Lamp2b-modified pEGFP-C1 vector to generate RVG-engineered EVs [112]. However, the genetic modification of EVs parental cells faces numerous difficulties, as the introduced surface-targeting ligands may affect the normal function of EVs membranes and introduce foreign impurities, resulting in lower EVs purity. This underlines the need for improved genetic engineering techniques for targeting parental cells.

#### Metabolic engineering

Besides genetic engineering, metabolic engineering can also be used to modify EVs parental cells by adding specific substances, such as amino acids, lipids, and polysaccharides, to the parental cell growth medium to promote cellular biosynthesis. After uptake, these substances are integrated into the proteome, glycoproteins, and liposomes contained by the EVs [109]. The metabolic labeling of newly synthesized proteins or glycan/glycoproteins of EVs-secreting cells has been combined with chemically active azide groups and bio-orthogonal click conjugation to modify and functionalize EVs [113].

#### Loading of exogenous substances

Modifications can also be accomplished by adding contents to parental cells through specific means. Tumor-targeted EVs loaded with miR-199a were obtained by the transfection of a specific binding transcriptional activator fused to Lamp2a into HEK293 cells, followed by incubation under suitable conditions [114].

### EVs engineering techniques: conclusions

EVs engineering can improve EVs tumor targeting ability and drug loading efficiency. For instance, azide-modified EVs generated from M1 macrophages were coupled with dibenzocyclooctyne-modified CD47 and SIRPα antibodies (aCD47 and aSIRPα). This modified EVs actively targeted tumors and blocked SIRPα and CD47 on macrophages, eliminating the “don't eat me” signal and improving macrophage phagocytosis [106]. However, complicated engineering processes restrict the therapeutic deployment of manufactured EVs on a wide scale, highlighting the need for the development of a simple and effective means of engineering EVs for improved tumor therapy. As an example, the development of electroporation and ultrasound devices, which are now widely used, has improved the efficiency of encapsulation of antitumor drugs.

## EVs storage

EVs stability varies under different storage conditions, such as different temperatures. EVs are usually stored at 4, − 20, or − 80 °C. Furthermore, protease inhibitors and alginose are added to the storage medium to safeguard the integrity of the EVs membrane [115, 116]. The protein content on the surface of EVs is highest when EVs are stored at 4 °C for a short time (24–48 h), while stability is greatest when they are stored at − 80 °C for a long time (> 1 week) [117]. Hence, the objectives of a given study and the storage period determine the best storage conditions for EVs. For short-term storage, isolated EVs can be maintained at 4 °C for days or weeks [116]. For months of storage, isolated EVs should be stored at − 80 °C. Furthermore, freezing and thawing cycles can significantly affect EVs durability. Studies have suggested that EVs are structurally susceptible to repeated freeze–thaw cycles owing to the vulnerability of their phosphatidylserine moieties [118]. Consequently, multiple freezing and thawing cycles should be avoided when storing EVs as this may greatly impair EVs integrity and stability [116].

## Tumor targeting with EVs

EVs may be utilized to address the poor tumor-targeting ability of traditional chemotherapeutic drugs. For instance, MSCs have homing capacity, that is, under the action of a variety of factors, they can cross vascular endothelial barriers to reach and colonize target tissues [119]. Furthermore, following ischemia, hypoxia, or injury, MSC-derived EVs (MSC-EVs) can migrate to sites of inflammation as well as to tumor tissues. This homing property of MSCs has been used to target MSC-EVs to tumor tissue and improve the efficiency of targeted tumor therapy. For example, the ability of MSC-EVs to target 5-fluorocytosine and mRNA to tumor tissues has been investigated for the development of new targeted therapies [120]. EVs secreted by tumor cells (Tu-EVs) may be able to act on tumor tissues. Their surface is decorated with parent cell-derived signaling molecules and their intravesicular content, including DNA, mRNA, miRNA, enzymes, and soluble factors, are all biologically active and capable of executing functional responses in target cells and retargeting to maternal tumor cells [121]. Tu-EVs carrying the chemotherapeutic drug doxorubicin can preferentially fuse with maternal cancer cells, thereby prolonging the retention time of the drug in the tumor [122]. This demonstrates the homing properties of Tu-EVs and provides a new direction for targeted tumor therapy. Additionally, biocompatible tumor-cell-exocytosed EVs-biomimetic porous silicon nanoparticles have been developed as drug carriers for targeted chemotherapy [123]. Therefore, the use of Tu-EVs offers promise for the targeted treatment of cancer.

The folate receptor (FR) is a glycoprotein present on the surface of cell membranes and is anchored to the cell membrane by GPI. It has a very high affinity for folate and is used for specific binding. FR is highly expressed in tumor cells, especially those of epithelial origin (e.g., pancreatic cancer) [124]. Thus, FR can be used to target tumors, while folate can be employed as a target for the preparation of FR-mediated tumor cell-targeted EVs (Co-EVs-FA) to increase the delivery of tumor-targeting drugs. For example, EVs-PH20-FA obtained by assembling folate into EVs through genetic engineering technology were shown to be able to target tumor tissue to enhance drug delivery [125].

Hyaluronic acid (HA), a glycosaminoglycan and a major component of the extracellular matrix, is highly expressed in several solid malignancies [126]. Hyaluronidases (HYALs) are endo-β-N-acetylglucosaminidases that degrade HA via hydrolysis of the β(1,4)-glycosidic bond between D-glucuronic acid and N-acetyl-D-glucosamine [127]. Therefore, mutual HYAL/HA recognition can be used to develop new strategies for tumor-targeting therapy. HA is highly concentrated in pancreatic ductal adenocarcinoma and is associated with a poor prognosis. The PEGylated form of recombinant human hyaluronidase PH-20 (PEGPH20) was used in pancreatic ductal adenocarcinoma alongside cytotoxic agents to enhance tumor targeting and the efficiency of drug delivery by recognizing HA around the tumor stroma [128].

Numerous other substances similar to FRs and HYALs, such as matrix metalloproteinases (MMPs), can be used in tumor-targeted therapy. Zhang et al. loaded MMP substrate peptide into multifunctional mesoporous SiO_2_ nanoparticles to selectively target MMP-rich hepatocellular carcinoma (HCC) cells and subsequently induce the intracellular release of a cytotoxic drug [129].

## Engineered EVs in tumor therapy

As drug carriers and delivery vehicles, EVs offer the following advantages: (1) Biological stability and the ability for long-term storage while maintaining their activity, thereby simplifying storage and transport; (2) low immunogenicity, which avoids activation of the immune response; (3) good biocompatibility, with almost no elimination by the immune system; and (4) a lack of differentiation ability, which prevents abnormal differentiation and tumor transformation [130–132]. Because EVs have a lipid bilayer membrane structure, their internal cavity can store water-soluble drugs, while the hydrophobic region in the lipid bilayer can receive hydrophobic drugs. This protects the contents from the effects of the harsh TME, thus preventing the decomposition of the drug before it reaches the tumor, as well as ensuring drug efficacy and the avoidance of toxicity [4]. In solid tumors, EVs have demonstrated enhanced permeability and a retention effect, resulting in targeted aggregation. The special membrane structure of EVs allows their direct fusion with the cell membrane and the transfer of the loaded drug into target cells, avoiding the problems of drug release and cytotoxicity associated with the phagocytosis-lysosome pathway [123]. These characteristics make EVs an ideal delivery system (Table 5).

**Table 5:** Therapeutic antitumor effects of various engineered extracellular vehicles (EVs)

Source of EVs	EV cargos	Loading approach	Type of cancer	Function	References
HEK293T cells	siRNA	Membrane-anchoring	Prostate cancer	Enhanced EV tumor targeting and inhibition of prostate cancer growth	[213]
HepG2 cells	miR-31 and miR-451	Electroporation	Hepatocellular carcinoma	Inhibition of tumor cell proliferation and migration	[214]
Panc02 cells	Nanoscale metabolic precursors	Click chemistry	Pancreatic cancer	Improving the tumor targeting of EV drug delivery systems	[215]
LNCaP PC-3 cells	PTX	Incubation	Prostate cancer	Improving chemotherapeutic drug delivery efficiency and enhancing cytotoxic effects	[134]
Macrophage cells	DOX, PTX	Electroporation/sonication	Lung cancer	Improving chemotherapy drug delivery efficiency to inhibit tumor growth	[91]
Mesenchymal stem cells	PLK-1 siRNA	Electroporation	Bladder cancer	Improving the targeting of EVs to tumor cells	[216]
Ovarian cancer	DOX	Electroporation	Ovarian cancer	Improving chemotherapy drug delivery efficiency to inhibit tumor growth	[217]
Red blood cells	Cas9 mRNA	Electroporation/incubation	Breast cancer	Improving the targeting of EVs to tumor cells and reducing adverse effects	[144]

### EVs loaded with different drugs for tumor therapy

Although chemotherapy is the mainstay of cancer treatment, side effects have limited its application. However, research into the targeting ability and delivery efficiency of EVs may provide a reference for improving their antitumor effect and mitigating the side effects associated with chemotherapy. Based on the excellent properties of EVs, multiple drugs have been loaded into EVs for tumor therapy, including chemotherapeutic drugs, RNAs, proteins, and even viruses.

PTX, which induces mitotic arrest, is used for the treatment of a variety of tumors [133]. Cancer cell-derived EVs loaded with PTX demonstrated enhanced prostate cancer targeting and cytotoxicity toward tumor cells [134]. Additionally, doxorubicin-loaded EVs were encapsulated with A33 antibody (A33Ab-US-EVs/Dox), which increased the ability of the EVs to target doxorubicin to colon cancer cells when compared with that for free doxorubicin, thereby extending survival in mice [135]. DNA replication, an essential process for tumor cell growth, can be disrupted by apolipoprotein-containing fibroblast-derived EVs loaded with methotrexate, which show excellent glioma targeting and killing effects [136]. Similarly, other chemotherapeutic agents, such as gemcitabine and cisplatin, have also been loaded into EVs for tumor-targeted therapy, and have demonstrated good effects [137].

RNAs can interfere with normal tumor metabolism by silencing gene expression in a sequence-specific manner. Thus, RNA-loaded EVs may provide options for tumor therapy. Mutations in the *KRAS* gene, which encodes a GTPase, are key drivers of pancreatic cancer [138]. SiRNAs that target oncogenic KRAS^G12D^ were loaded into normal fibroblast-derived EVs by electroporation, and these engineered EVs targeted pancreatic cancer tissue and inhibited tumor-cell growth [139]. Resistance to chemotherapeutic drugs is an ongoing challenge in tumor treatment. Recently, it was found that miRNAs can be used to modify the drug resistance phenotype of tumor cells. An in vitro assay showed that EVs derived from human gastric epithelial cells could deliver anti-miR-214, resulting in the reversal of chemoresistance to cisplatin in gastric cancer cells [140]. Genetic engineering is an emerging therapeutic modality for tumors. MiRNAs have been shown to regulate tumor cell migration, invasion, and pre-metastatic niche formation through the transcriptional repression of target genes. For instance, ovarian cancer cell-derived EVs loaded with miR-199a-3p could downregulate the expression of the miR-199a-3p target gene mesenchymal-epithelial transforming factor (c-Met), which inhibited tumor cell proliferation and invasion [141]. Engineered EVs were used to deliver the antitumor drug fluorouracil (5-FU) and miR-21 inhibitor oligonucleotides to colon cancer cells expressing human epidermal growth factor receptor-2 (HER2), which reversed the resistance of the cancer cells to 5-FU and enhanced antitumor cytotoxicity [142]. These studies highlight the potential of RNA-loaded EVs for engineering applications in the treatment of cancer.

The CRISPR/Cas 9 system, widely used as a genome editing tool, holds excellent promise in biomedicine, especially for drug delivery in oncology. In one study, ovarian cancer-derived EVs were loaded with plasmids expressing CRISPR/Cas9 targeting poly (ADP-ribose) polymerase-1 (PARP-1) to treat ovarian cancer. This engineered EVs system synergized with cisplatin to induce apoptosis in ovarian cancer cells [143]. Similarly, red blood cell-derived EVs were loaded with Cas9 mRNA targeting miR-125b, which enabled gene editing and, consequently, the inhibition of leukemia cell growth through the suppression of miR-125b expression [144].

The emergence of viral therapies may offer new options for oncological therapy. Oncolytic viruses can be engineered to selectively infect tumor cells without affecting normal tissue. They replicate in the cancer cells and eventually cause their death [145]. In a lung cancer-related study, oncolytic viruses and PTX were encapsulated in lung cancer cell-derived EVs, which were then administered intravenously for tumor treatment. This engineered EVs system demonstrated stronger tumor growth inhibitory activity compared with free PTX [146]. Collectively, these different agents provide new paradigms for EVs-based oncological therapy.

### EVs from different cells used in tumor therapy

**Fig. 2:**
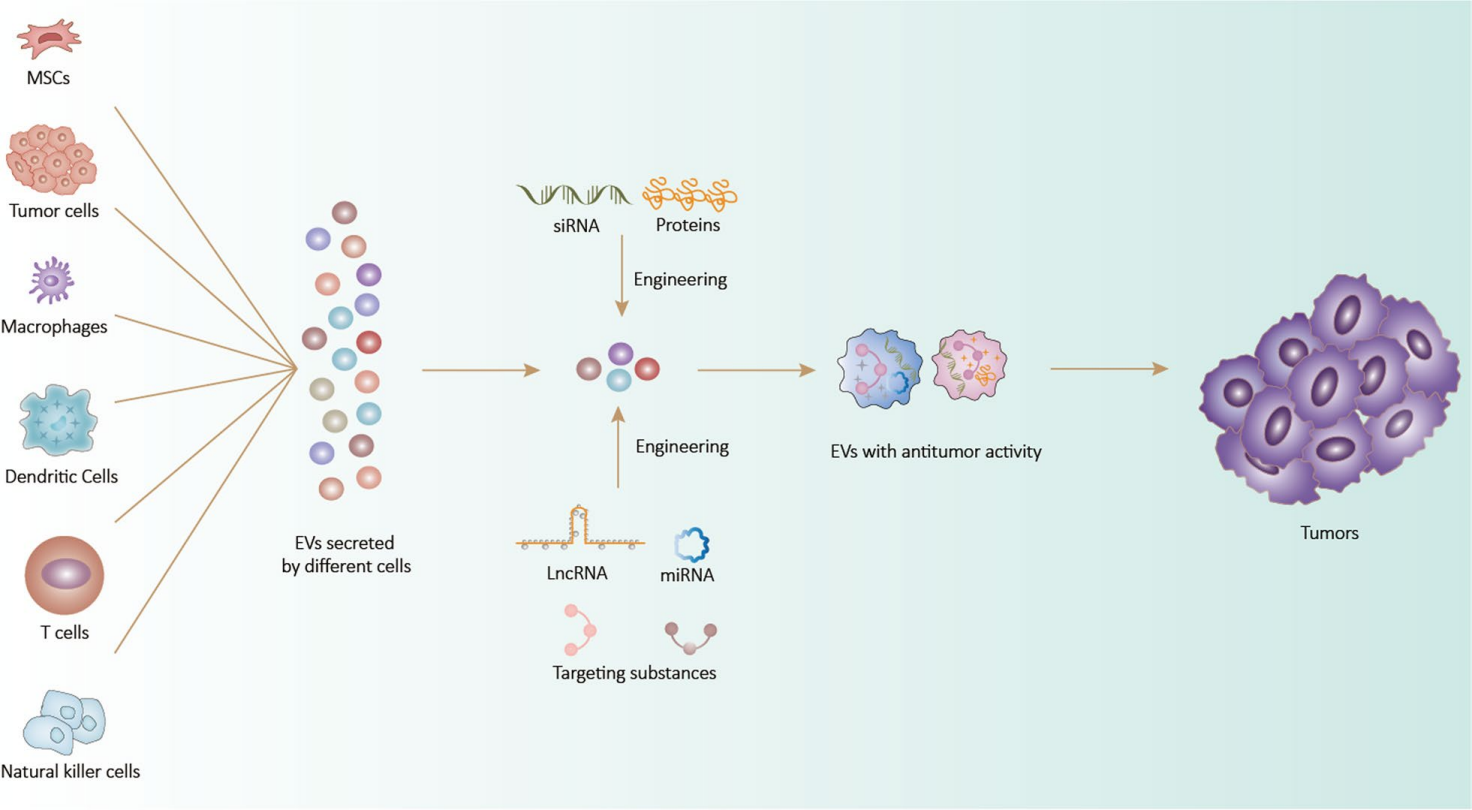
After being engineered, extracellular vesicles (EVs) secreted by different cells exert therapeutic effects on tumors

Due to the multiple substances carried by EVs, MSCs-EVs can play an important role in cell proliferation and immune regulation and affect tumor progression by regulating the TME [147]. Although the conventional view holds that MSC-EVs can promote tumor growth and metastasis [148], many studies have suggested that some MSC-EVs can exert the opposite effects. Furthermore, mounting evidence indicates that MSC-EVs also have strong therapeutic potential, e.g., for repairing tissue, eliminating inflammation, regulating immunity, and suppressing tumors [149]. Importantly, MSCs have a strong capacity for EVs production and MSC-EVs have tumor-homing ability [119]. Based on these characteristics, MSC-EVs show great promise as tools for tumor-targeting therapy. Numerous studies have shown that MSC-EVs loaded with antitumor drugs can specifically target tumor tissues [150], resulting in stronger antitumor effects and fewer side effects. For instance, EVs derived from bone marrow MSCs (BM-MSC-EVs) loaded with doxorubicin were reported to improve the efficiency of drug uptake and antitumor effects in MG63 osteosarcoma cells [151]. Similarly, modified MSC-EVs expressing miRNA-199a, which targets AGAP2, were delivered to glioma cells, resulting in the inhibition of glioma development [152]. Immune escape is a key factor in tumor growth and invasion. Some MSC-EVs can inhibit tumor progression by suppressing immune escape in the TME, and generating drug delivery systems using this mechanism may further improve the efficiency of tumor therapy. For example, EVs from adipose tissue-derived MSCs (AD-MSCs) modified to express miRNA-424 can delay the progression of triple-negative breast cancer by blocking programmed cell death protein 1/programmed cell death 1 ligand 1 (PD-1/PD-L1) [153]. Furthermore, the loading of siRNA and oxaliplatin into BM-MSCs-EVs by electroporation can enhance the antitumor effect of oxaliplatin in pancreatic ductal adenocarcinoma [154]. This could be achieved by increasing the recruitment of T lymphocytes and downregulating that of regulatory T cells (Tregs), thereby suppressing immune escape. We suggest that using the MSC-EVs-based drug delivery system with tumor-suppressive capacity may represent a feasible strategy for tumor immunotherapy (Fig. 2).

EVs secreted by tumor cells are involved in a variety of cellular functions and pathological events in the TME. Tu-EVs carry a variety of miRNAs and mRNAs that can be transferred to recipient cells, with the latter being translated into functional proteins [155]. Accordingly, Tu-EVs can support cancer transformation by delivering oncogenic signals to target cells, thus maintaining the autocrine growth-promoting pathway in parental tumor cells and altering stromal cell function in the TME [156]. Importantly, because Tu-EVs carry the same cytokines or chemokines as parental cells, signal delivery occurs preferentially between parental tumor cells [157]. Hence, Tu-EVs can be used as drug carriers in oncology owing to their excellent targeting capability with respect to parental cells. For instance, breast cancer cell-derived EVs loaded with doxorubicin can target breast cancer cells and inhibit tumor invasion by suppressing tumor angiogenesis [158]. Moreover, EVs derived from B16 melanoma cells transfected with a plasmid encoding the *Mycobacterium tuberculosis* antigen early secretory antigenic target-6 (ESAT-6) were found to be able to significantly inhibit tumor growth in syngeneic B16 tumor-bearing mice [159]. These observations highlight the great potential of Tu-EVs as a drug carrier; however, future research should focus on how to avoid events where substances carried by Tu-EVs can promote tumor progression.

EVs derived from immune cells can target tumor cells, thus mimicking the parent cell, and exhibit drug-carrying capacity. In recent years, EVs secreted by macrophages (M-EVs) have been modified for tumor therapy, with promising results. The loading of PTX into M-EVs by ultrasonication led to a 50-fold enhancement of the cytotoxic effect of PTX on drug-resistant tumor cells [91]. Similarly, Li et al. modified M-EVs with a peptide to target c-Met to increase their tumor-targeting capability, and these modified M-EVs were coated onto a poly(lactic-co-glycolic acid) nanoplatform for delivery to triple-negative breast cancer. This modified M-EVs system significantly improved the cellular uptake efficiency and antitumor efficacy of doxorubicin [160]. DC-derived EVs (DC-EVs) contain co-stimulatory molecules, integrin avβ2, intercellular adhesion molecule 1 (ICAM1), and other components involved in cell–cell interactions [161], which allows DC-EVs to control tumor immune escape by modulating immune stimulation, thereby inhibiting tumor progression [162]. This property of DC-EVs has been investigated to load drugs to enhance antitumor effects. For instance, the loading of fetoprotein into DC-EVs induced antigen-specific immune responses, including the upregulation of IFN-γ and IL-2 and the downregulation of Tregs recruitment, which inhibited HCC growth [163]. In addition, EVs purified from DCs loaded with antigens and matured with the Toll-like receptor 3 (TLR3) ligand poly (I:C) strongly stimulated the proliferation of CD8^+^ and CD4^+^ T cells and recruited CD8^+^ T cells and natural killer (NK) cells to tumors, thereby enhancing the antitumor functions of the EVs and inhibiting melanoma growth [164]. Meanwhile, EVs derived from T cells and NK cells also inhibited tumor progression by responding to specific antigens and producing cytokines that modulated the immune response. EVs derived from genetically engineered chimeric antigen receptor (CAR)-T cells express CAR, CD63, CD3, and C-X-C motif chemokine receptor 4 (CXCR4), which allows them to exert potent antitumor effects [165]. Moreover, EVs secreted by NK cells (NK-EVs) can exert cytolytic effects on tumor tissues [166], while miRNA-loaded biomimetic core–shell nanoparticles that act together with modified NK-EVs increased drug targeting and miRNA delivery efficiency in neuroblastoma cells, resulting in the dual inhibition of tumor growth [167].

### EVs-based drug co-delivery system

**Fig. 3:**
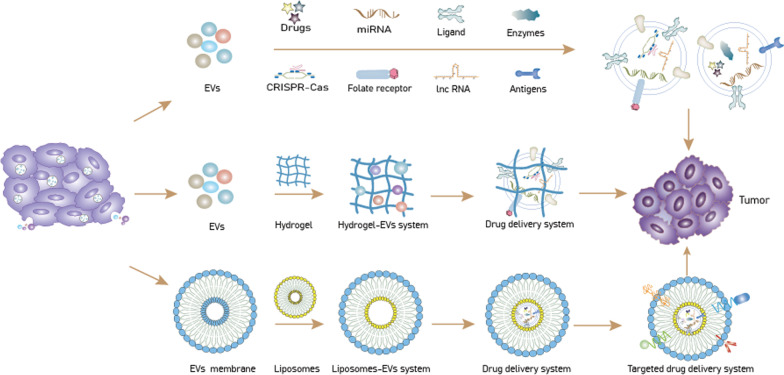
Targeted tumor therapy based on extracellular vesicle (EVs)/hydrogel or EVs/liposome co-delivery systems

To further enhance the delivery effect of EVs, research attention has increasingly focused on the development of EVs-nanodrug co-delivery systems (Fig. 3). These systems can enhance drug delivery efficiency by combining the advantages of EVs and those of inorganic nanocarriers. Combining EVs with hydrogels and liposomes is increasingly common. Compared with existing stand-alone inorganic nanocarriers such as gold nanoparticles and therapeutic nano-bioconjugates, this combinatorial drug delivery approach improves EVs targeting and drug encapsulation efficiency [168–173]. Furthermore, the exceptional biocompatibility of the co-loading system renders it suitable for tumor-targeted therapy.

Hydrogels are three-dimensional network structures with good biocompatibility and high water content [174]. They can serve as drug carriers for tumor therapy as they have properties similar to those of the extracellular matrix [175]. However, the low stability, poor mechanical properties, and poor cell adhesion properties of natural hydrogels limit their application as drug carriers [176]. Synthetic hydrogels have received increasing attention based on the reliability of their sources, long shelf life, and low risk of immunogenicity [177]. The most commonly employed hydrogel synthesis system involves the incorporation of EVs. For example, in one system, thioglycolic acid, gelatin, and heparin were used as the main components and polyethylene glycol diacrylate (PEGDA) as the gelling agent; BM-MSCs-EVs were then incorporated into this polymer to complete the preparation of the drug delivery system [178]. In addition, the integration of EVs and their parent stem cells into hydrogels has been proposed as a means of achieving sustained EVs delivery [179]. Hydrogel-EVs systems have shown promise in tumor treatment. For instance, a hydrogel-EVs system developed for the delivery of doxorubicin and celecoxib showed good drug delivery efficiency and significantly inhibited tumor growth [180]. Additionally, the combined application of hydrogel and human umbilical cord MSC-EVs (hUC-MSC-EVs) to deliver drugs to sites of disease led to a significant increase in drug delivery efficiency, which provides guidance for tumor-targeted therapy despite this effect referring to a model of diabetes [181].

Liposomes are tiny vesicles enclosed in a membrane bilayer consisting mainly of phospholipids and cholesterol that can encapsulate drugs and serve as valuable drug delivery systems [182]. However, instability, lack of drug encapsulation repeatability, a lack of particle size control, and a short half-life are all factors that have hampered the development of liposome-based therapy [183–185]. EVs have many similarities with liposomes and these similarities allow for the use of liposome-related technology to design EVs, such as when EVs and liposomes are fused to construct a drug co-delivery system. Compared with stand-alone liposome drug delivery systems, liposome-EVs formulations exhibit greater anticancer efficacy owing to slower drug release and longer drug half-life [186, 187]. Moreover, the fusion of EVs with liposomes increases loading capacity while retaining the targeting ability of EVs [75]. In one study, the authors fused liposomes with MSC-EVs and obtained a hybrid liposome-EVs system that was then loaded with miR-34a. The system was shown to significantly enhance miRNA delivery efficiency and delay the progression of breast cancer [77]. Additionally, the synthesis of thermosensitive hybrid EVs-liposome nanoparticles loaded with doxorubicin led to a significant improvement in the efficiency of chemotherapeutic drug delivery and inhibited the growth of metastatic peritoneal cancer [188]. Surprisingly, the biocompatibility of the EVs prevents the drug co-delivery system from being eliminated by the immune system in vivo, hence retaining the stability of the drug delivery system. Microfluidic and lab-on-a-chip technologies have enabled the production of liposomes of controllable size using simple methods. Such uniform liposomes make the large-scale manufacture of EVs-liposome drug delivery devices possible [189]. Of note, the hydrophobicity of lipid molecules makes the fusion of liposomes and EVs difficult; accordingly, a new strategy for the preparation of liposome-EVs fusions has been proposed, namely, the use of freeze–thaw cycles [190]. Overall, liposome-EVs systems have excellent potential for targeted tumor therapy.

## Conclusions

Because of their excellent properties, EVs may be an ideal drug delivery vehicle for tumor therapy. The engineering of EVs for the treatment of tumors also shows promise and has been widely studied owing to its superiority over natural EVs. In this review, we discussed techniques commonly used for the isolation of EVs, specific EVs modifications, and the therapeutic effects of engineered EVs on tumors. The presented evidence highlighted the utility of using EVs for targeted tumor therapy. However, although EVs have been investigated in clinical trials, further research is warranted to confirm their excellent antitumor therapeutic effects. Owing to differences in the biological properties of EVs secreted by different cells, there is currently no unified standard for their isolation. In addition, the high cost and unsatisfactory isolation efficiency are currently the primary factors limiting large-scale EVs production. For instance, ultracentrifuge-based EVs isolation alone may suffice for preclinical analysis but not for real-world clinical application because of the presence of co-isolates. Furthermore, isolation techniques such as immunochromatography and ultrafiltration are time-consuming and are not suitable for large-scale sample isolation. Therefore, it is advisable to combine different isolation techniques to improve separation efficiency, such as ultracentrifugation to remove impurities followed by immunoaffinity chromatography. Microfluidic technology has been used to isolate and purify EVs from culture medium, and the structural integrity and functional stability of the isolated EVs were retained with this method. This approach is characterized by simplicity, high sensitivity, and low cost, showing excellent application potential. Nevertheless, the EVs isolation efficiency associated with this technique requires further improvement [191, 192].

For EVs modification, it is necessary to fully understand the composition of the EVs membrane at the target site, and select the appropriate targeting ligands. This is important to avoid changes in the charge on the EVs membrane during the modification process and maintain its physicochemical stability. Notably, genetic engineering can only be used to modify encoded proteins or polypeptides. When performing modifications at the genetic level, the functional stability of EVs membrane proteins must be maintained. The modification of EVs surface proteins using amino acids, lipids, and polysaccharides requires suitable chemical and physical treatment for a firm attachment. Because the treated EVs may contain high levels of impurities, EVs must be isolated and purified to ensure safety. Further research on EVs modification methods is warranted. For drug loading, chemotherapeutic agents are currently the primary choice, and engineering modifications has improved the targeting and drug loading efficiency of EVs. Furthermore, using engineered modifications, gene therapy also exhibits tumor-targeting therapeutic potential, highlighting the good application prospects of EVs loaded with RNAs, nucleic acids, and other contents. Despite these observations, pre- and clinical applications of engineered EVs face many challenges. Drug delivery efficiency, scalability, stability, and tumor targeting potential of engineered EVs require optimization, such as how to improve the targeting of EVs by chemical modification while maintaining their membrane integrity.

Co-delivery systems may be an option for further improving EVs drug delivery efficiency and tumor targeting abilities. In addition to hydrogels and liposomes, multiple inorganic nanocarriers, such as dendrimers and micelles, have also received attention for cellular targeting, high-dose drug delivery, and gene therapy payloads [193]. Relatively few studies have investigated EVs-based co-delivery systems given the associated limitations, which include the complexity of technical manipulation. However, these nanocarrier-EVs systems have great potential for drug delivery in oncology and merit further exploration. The effect of EVs secreted by different cells on tumor progression varies. Thus, selecting EVs with antitumor effects as drug carriers may improve the efficiency of antitumor therapy. MSCs have a robust EVs production capacity compared with most other cells [194], while the low immunogenicity of MSC-EVs allows them to avoid activating the immune response and, consequently, clearance by the immune system [195]. In addition, MSC-EVs have a unique homing ability, a property that can be harnessed for targeted tumor therapy. These advantages make EVs the primary choice as drug delivery systems for targeted tumor therapy. EVs secreted by other immune cells, such as M1-EVs, DC-EVs, and NK-EVs, can inhibit tumor progression by affecting interactions within the tumor microenvironment; accordingly, these EVs also have excellent potential as drug delivery systems.

In conclusion, the use of modified EVs has the potential to contribute greatly to tumor therapy. As the demand for precise tumor treatment increases, there is an urgent need to understand the mechanisms associated with EVs biogenesis and transport, as well as those involved in EVs signaling after modification. We hope that this review will promote the clinical application of EVs.

## Data Availability

Not applicable.

## Abbreviations

BM-MSCs-EVs: Extracellular vesicles derived from bone marrow mesenchymal stem cells
CCT2: Chaperonin containing TCP1 subunit 2
CXCR4: C-X-C motif chemokine receptor 4
DC: Dendritic cells
DC-EVs: Dendritic cells secreted extracellular vesicles
FBS: Fetal bovine serum
FR: Folate receptor
GPI: Glycosyl phosphatidyl inositol
HA: Hyaluronic acid
HCC: Hepatocellular carcinoma
HPL: Human platelet lysate
HS: Human serum
HYALs: Hyaluronidases
lncRNA: Long non-coding RNA
M-EVs: Extracellular vesicles from macrophages
miRNA: MicroRNA
MMP: Matrix metalloproteinases
MSCs: Mesenchymal stem cells
MSCs-EVs: Extracellular vesicles derived from mesenchymal stem cells
NK: Natural killer cells
NK-EVs: Natural killer cells secreted extracellular vesicles
PD-1: Programmed death-1
PD-L1: Programmed death-ligand 1
PEG: Polyethylene glycol
PTX: Paclitaxel
SIRPα: Signal regulatory protein alpha
Tu-EVs: Tumor-derived extracellular vesicles
Ti-EVs: Tissue-derived extracellular vesicles
UC: Ultracentrifugation
